# Investigating the Correlation Between Anterior-Posterior and Lateral Asymmetric Muscular Balance With Low Back Pain

**DOI:** 10.7759/cureus.9785

**Published:** 2020-08-16

**Authors:** Stephen Albano, Ruby Gilmor, Kevin Calvelo, Rehman Afraz, Mary Grace Bacani, Javed Siddiqi

**Affiliations:** 1 Neurosurgery, Desert Regional Medical Center, Palm Springs, USA; 2 College of Osteopathic Medicine, Touro University of California, Vallejo, USA; 3 Intensive Care Unit, Desert Regional Medical Center, Palm Springs, USA; 4 Neurological Surgery, Desert Regional Medical Center, Palm Springs, USA

**Keywords:** sagittal balance, low back pain, muscular balance

## Abstract

Objective

The objective of this pilot study was to determine if there is a correlation between the proposed physical testing protocol and low back pain. The proposed physical testing protocol is an attempt to assess muscular asymmetry in the anterior-posterior plane and the lateral plane.

Methods

A total of 96 volunteers were recruited from Touro University after obtaining IRB approval. Volunteers were initially provided a questionnaire regarding demographics and back pain. After ensuring participants satisfied the inclusion criteria, a physical test protocol was performed. After data compilation, odds ratios as well and linear regression models were generated to assess for correlation with back pain.

Results

A total of 96 participants were recruited. The odds ratio for asymmetric anterior-posterior balance in relation to back pain is 3.00 with a 95% confidence interval 1.26-7.12. The odds ratio for total ability to tolerate asymmetric loads greater than 50% of ideal body weight is 0.44 with a 95% confidence interval 0.11-1.77. The linear regression coefficient of anterior-posterior balance greater than 25% of ideal body weight in relation to level of pain is 1.96.

Conclusions

Increased muscular asymmetry in the sagittal plane and lateral plane showed a trend toward increased levels of low back pain; however, there is a weak correlation. This is a correlation and not an association. Future studies to assess the relationship between muscular balance and low back pain are needed to determine if therapy can be targeted to improve muscular sagittal balance, which can improve symmetry and back pain.

## Introduction

Low back pain is a common problem in the medical community prompting visits to emergency rooms, primary care physicians, pain management specialists, orthopedists, and neurosurgeons. There are multiple etiologies ranging from emergencies like traumatic fractures to strains. Low back pain “is the leading cause of activity limitation and work absence” [1], and it affects four out of five adults at some point in their lives [2,3]. In 2010, the prevalence in a three-month period was found to be approximately 25% [4]. The workup and management of low back pain have caused the economic burden to raise exceeding $100 billion per year in 2006 [5]. This paper is a prospective pilot study in measuring sagittal and coronal muscular asymmetry and assessing if there is a correlation with low back pain.

## Materials and methods

A pilot case-control study was performed. Participants were recruited from volunteers at a medical school after obtaining IRB approval. Participants were asked to respond to a questionnaire regarding gender, age, weight, height, and specifics regarding low back pain severity on the visual analog scale within the past week. Low back pain was defined as the pain in the area between right and left posterior superior iliac spine and sacral ala and lowest ribs. Patients less than 18 years old, who were pregnant, who underwent low back surgery, or who were scheduled to undergo low back surgery were excluded from the study. Participants who rated back pain as an 8 or greater in the past week were also excluded to prevent possible exacerbation of pain due to study protocol.

The authors developed the physical testing protocol used in the study. The muscular balance was then assessed by having the patient stand on a single leg on an elevated surface (at least 2 inches off the ground but not more than 6 inches) holding a 5 pound (lb) weight in the contralateral hand for 30 seconds. If the patient successfully maintained a balance on a single leg without contralateral leg touching the ground then the process was repeated using a 10 pound (lb) weight. The process was repeated with incremental increases in weight by 5 pounds (lbs) until failure or until 50 pounds (lbs) was reached. Then, the process was once again performed on the opposite leg. The leg and failure weight or max 50 pounds (lbs) weight was recorded for each participant.

Participants were then asked to lay supine with hips on a flat elevated surface (at least 2 inches and not more than 6 inches off the ground). The patient was then asked to elevate legs, upper torso, and arms off of the ground while balancing on the elevated surface. The referenced position is a yoga pose called boat pose. Arms could be placed in a position of comfort or to help maintain balance as long as no part of the body was touching the ground. Time of elevation until any body part touched the ground was recorded or until a maximum of three minutes was reached. The participant was then asked to flip over into a prone position with sacrum on an elevated surface. The participant was asked to elevate legs, upper torso, and arms off the ground (assuming an arched back position). Time to failure where any body part touched the ground or until a maximum of three minutes was recorded.

Data were analyzed using odds ratio and linear regression models to determine if a correlation existed between low back pain and different risk factors, primarily muscular symmetry. Definition of risk factors based on null hypotheses and definition of which participants were placed in which groups are shown in Table 1.

**Table 1:** Definition of groups and null hypotheses R = maximum weight in kg participant able to hold in left hand for 30 seconds while standing on right single leg L = maximum weight in kg participant able to hold in right hand for 30 seconds while standing on left single leg A = maximum time in seconds patient able to hold abdominal contraction to keep legs and arms off ground when starting in supine position P = maximum time in seconds patient able to hold back arch to keep legs and arms off ground when starting in prone position IBW = ideal body weight IBW (males) = 50 kg + 2.3 kg (height in inches – 60 inches) IBW (females) = 45.5 kg + 2.3 kg (height in inches – 60 inches)

Null hypothesis	Exposed (n who meet criteria)	Unexposed (n who meet criteria)
Asymmetric lateral muscular balance does not correlate with back pain	(|R-L|)/IBW > 25%	(|R-L|)/IBW < 25%
Asymmetric anterior-posterior muscular balance does not correlate with back pain	(|A-P|)/IBW > 25%	(|A-P|)/IBW < 25%
Total ability to tolerate lateral asymmetric loads does not correlate with back pain	(R+L)/IBW > 50%	(R+L)/IBW < 50%
Total ability to tolerate anterior-posterior asymmetric loads does not correlate with back pain	(A+P)/IBW > 50%	(A+P)/IBW < 50%

The basis for the linear regression model is shown in Table 2. 

**Table 2:** Linear regression models

Linear regression models	X-axis	Y-axis
Asymmetric lateral/coronal muscular balance does not correlate with back pain	x = (|R-L|)/IBW	Pain scale from 0-10 (8-10 excluded from the study to prevent injury from study protocol)
Asymmetric anterior-posterior/sagittal muscular balance does not correlate with back pain	x = (|A-P|)/IBW	Pain scale from 0-10 (8-10 excluded from the study to prevent injury from study protocol)
Total ability to tolerate lateral/coronal asymmetric loads does not correlate with back pain	x = (R+L)/IBW	Pain scale from 0-10 (8-10 excluded from the study to prevent injury from study protocol)
Total ability to tolerate anterior-posterior/sagittal asymmetric loads does not correlate with back pain	x = (A+P)/IBW	Pain scale from 0-10 (8-10 excluded from the study to prevent injury from study protocol)

## Results

A total of 96 participants were included in the study. Participants had an average age of 25 years with 64.6% of them being male. The average height of participants was 68 inches with an average weight of 161 pounds.

Based on groupings as defined in Table 1, participants were assigned to groups shown in Table 3, and odds ratios with 95% confidence intervals were calculated. The linear regression coefficients for the groups’ respective asymmetry to pain level are shown in Table 4. The odds ratio for asymmetric lateral muscular balance >25% in relation to low back pain was unable to be assessed due to some groups having zero participants meeting those criteria. Similarly, no odds ratio for total anterior-posterior load tolerance in relation to low back pain could be generated due to a denominator of zero since there were no participants with back pain whose total anterior-posterior tolerance normalized to ideal body weight was less than 50%. The odds ratio for an asymmetric anterior-posterior balance greater than 25% in relation to back pain was 3.00. The odds ratio for total lateral asymmetric load tolerance in relation to low back pain is 0.44.

**Table 3:** Distribution of participants for odds ratio

Null hypothesis	Exposed no pain	Exposed with pain	Unexposed no pain	Unexposed with pain
Asymmetric lateral muscular balance does not correlate with back pain	0	0	60	36
Asymmetric anterior-posterior muscular balance does not correlate with back pain	24	24	36	12
Total ability to tolerate lateral asymmetric loads does not correlate with back pain	56	31	4	5
Total ability to tolerate anterior-posterior asymmetric loads does not correlate with back pain	59	36	1	0

Linear regression models were generated and the coefficient of the best fit line is shown in Table 4.

**Table 4:** Odds ratios with 95% confidence intervals, linear regression coefficient, and R2 value

Null hypothesis	Odds Ratio	Confidence interval (95%)	Linear regression coefficient	R^2^ value
Asymmetric lateral muscular balance does not correlate with back pain	Unable to assess	Unable to assess	31.8	14%
Asymmetric anterior-posterior muscular balance does not correlate with back pain	3.00	1.26-7.12	1.96	17%
Total ability to tolerate lateral asymmetric loads does not correlate with back pain	0.44	0.11-1.77	-5.81	8%
Total ability to tolerate anterior-posterior asymmetric loads does not correlate with back pain	Unable to assess	Unable to assess	-0.11	<1%

**Figure 1:**
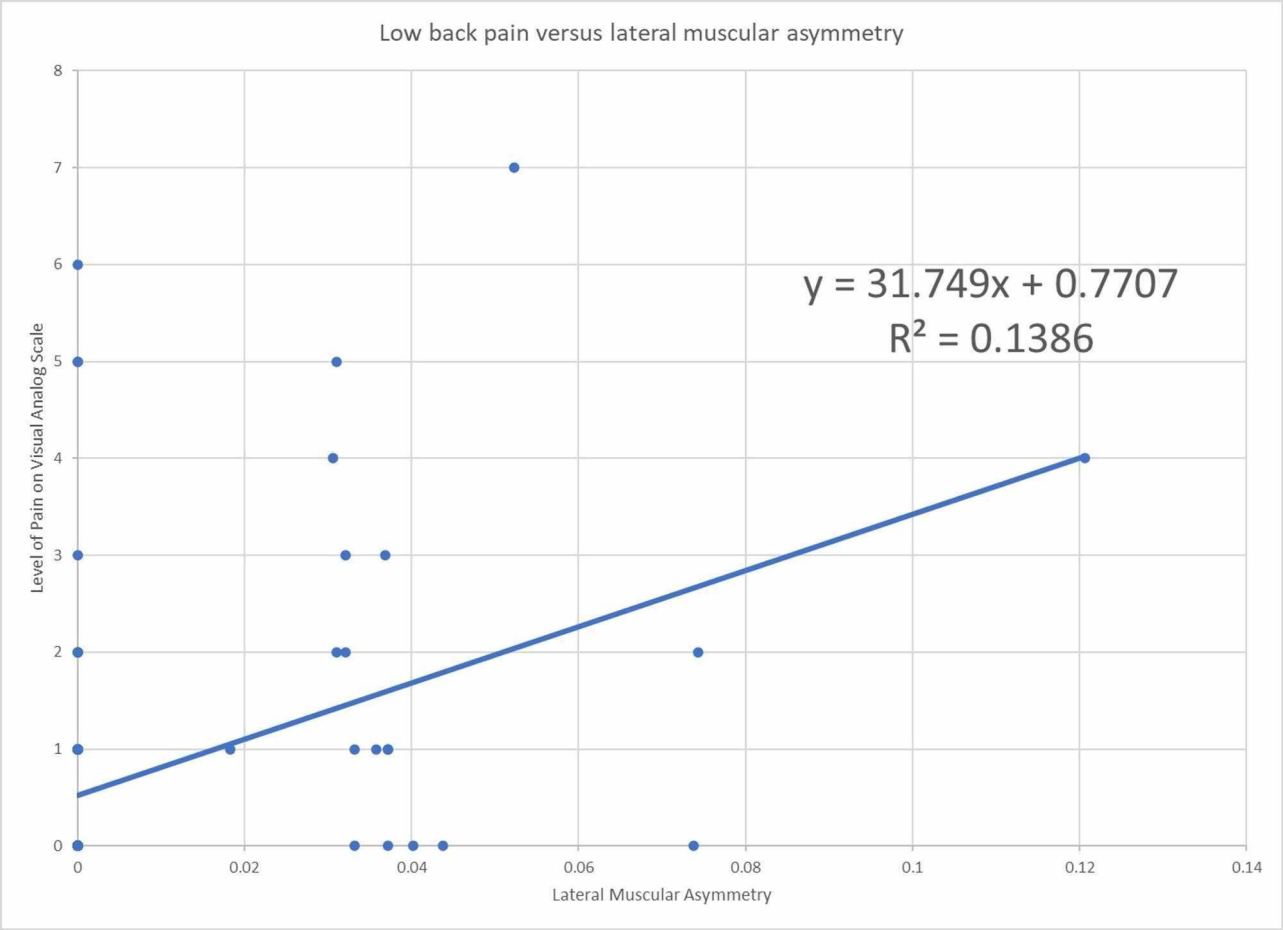
Low back pain as a function of lateral muscular asymmetry normalized to ideal body weight

Figure 1 shows data points and linear regression best fit line for asymmetric lateral balance to the level of back pain in the past week with a coefficient of 31.8 and R^2^ value 14%. 

**Figure 2:**
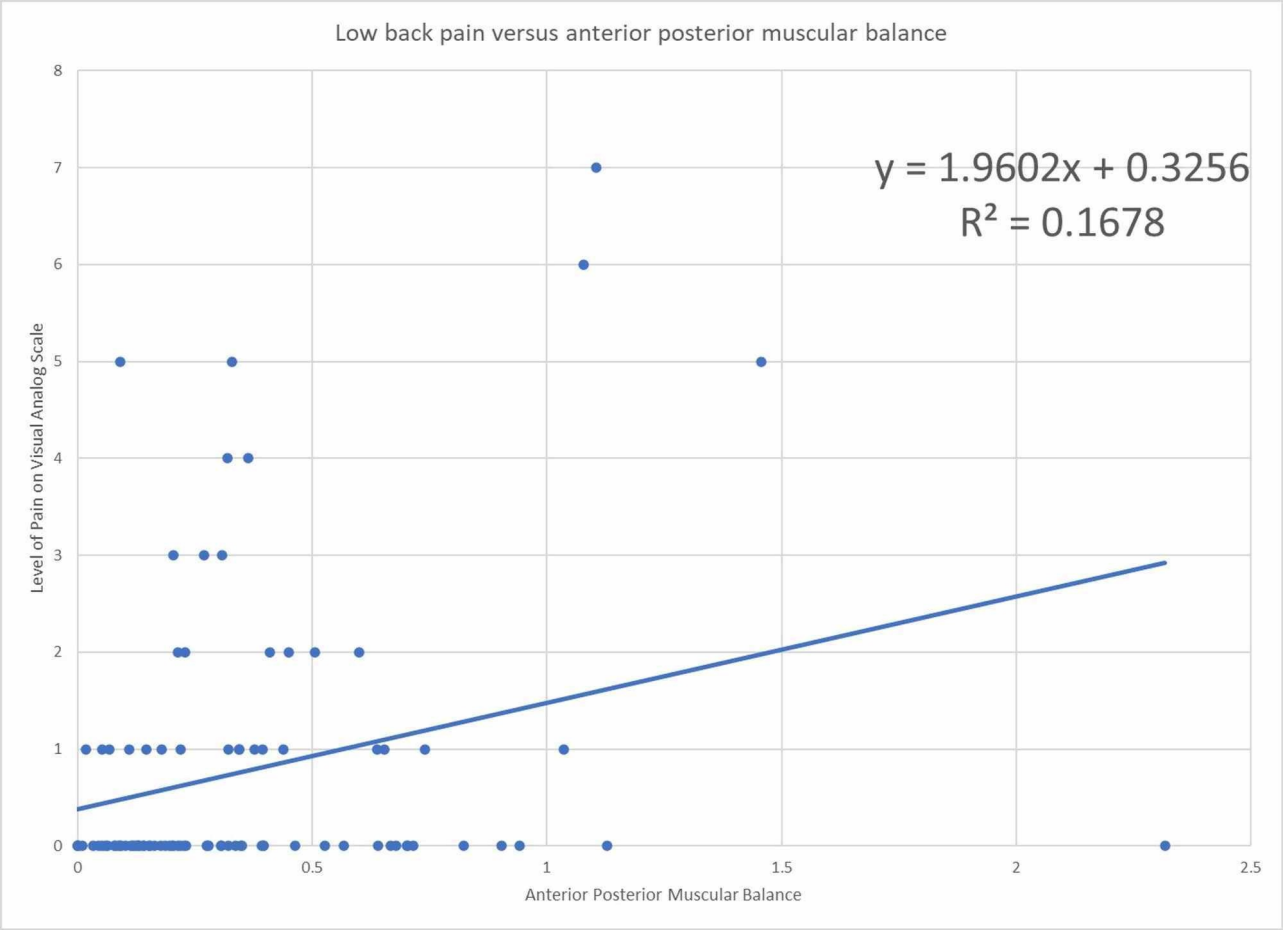
Low back pain as a function of anterior-posterior muscular asymmetry normalized to ideal body weight

Figure 2 shows data points and linear regression best fit line for asymmetric anterior-posterior muscular balance to the level of back pain in the past week with R^2^ value 17%. 

**Figure 3:**
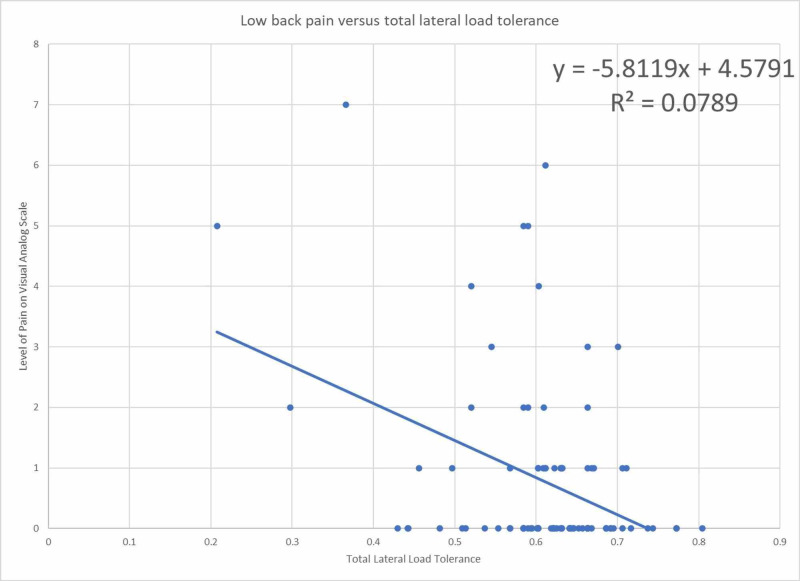
Low back pain as a function of total lateral load tolerance normalized to ideal body weight

Figure 3 shows the total ability to tolerate lateral asymmetric loads to the level of back pain with an R^2^ value of 8%. 

**Figure 4:**
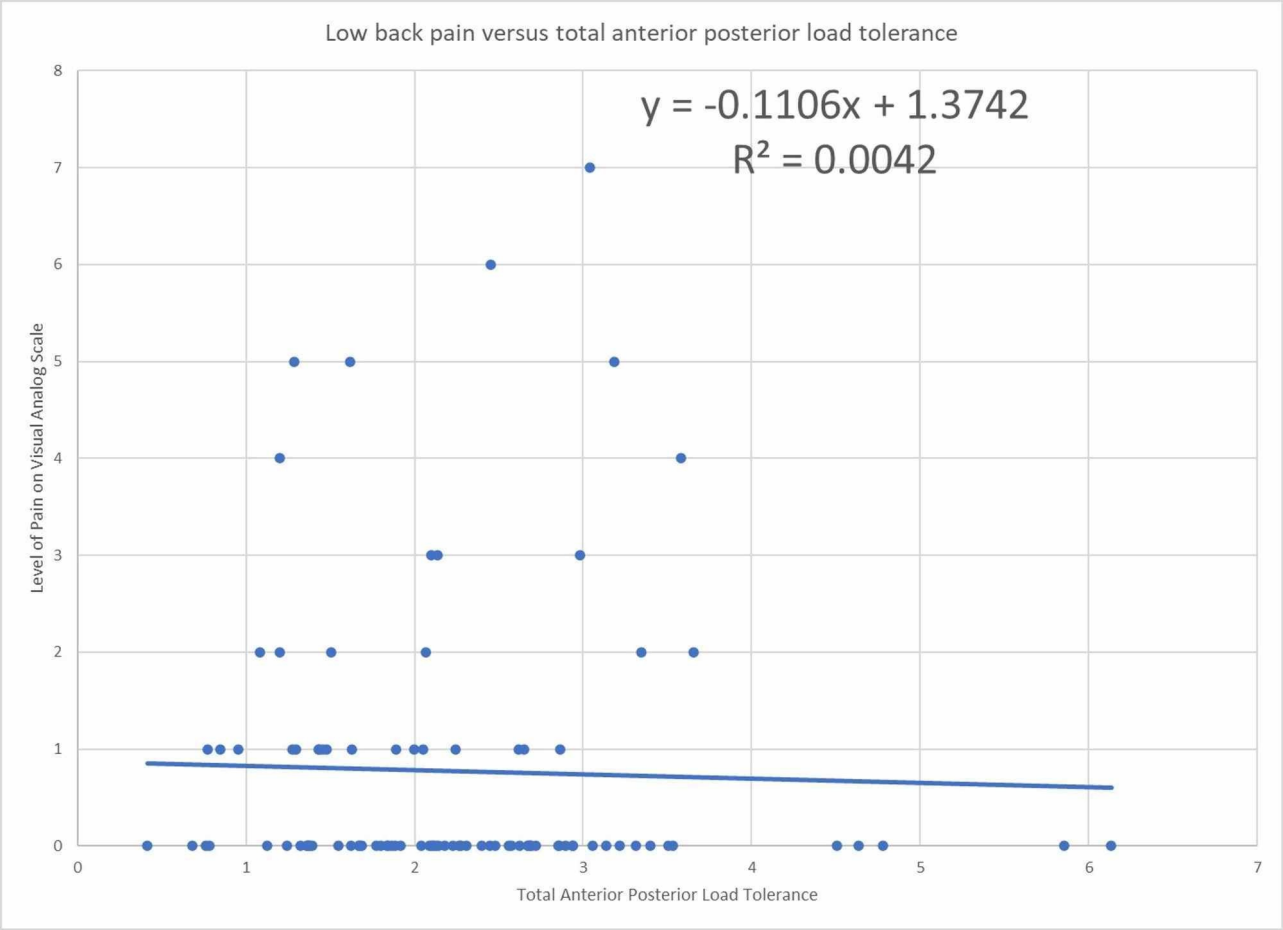
Low back pain as a function of total anterior-posterior load tolerance normalized to ideal body weight

Figure 4 shows the total ability to tolerate anterior-posterior loads to the level of back pain with an R^2^ value of less than 1%. 

## Discussion

The odds ratio for having an asymmetric lateral balance with low back pain was unable to be assessed. There were no participants with asymmetry greater than 25% of ideal body weight. This cutoff was created prior to data collection and therefore was not changed after completion to maintain prospective analysis. Due to the binary nature of odds ratio, a linear regression model was indicated by the degree of lateral asymmetry on the x-axis and the level of pain ranging from 0 to 10 on the y-axis to see if there was a correlation. The linear regression best fit line has a coefficient of 31.8 with a positive correlation indicating that as asymmetry increased, the level of back pain also increased. However, the R^2^ value for low back pain in relation to lateral muscular asymmetry is 14% which indicates a weak strength of the relationship between the variables. This finding coincides with prior studies that suggest postural control is not associated with back pain [6-9]. However, the positive coefficient of the best fit line and low R^2^ value demonstrated in this study contrasts a study showing poorer postural control in patients with back pain [10].

The odds ratio for anterior-posterior muscular imbalance in the setting of low back pain is 3.0 with 95% confidence intervals that reject the null value. This suggests that low back pain correlates with anterior-posterior muscular balance. The linear regression model also demonstrates a positive linear regression coefficient 1.96 indicating that as imbalance increases, low back pain also increases. However, the R^2^ value is 17% indicating a weak relationship between variables. This weak relationship is likely due to the multifactorial nature of low back pain. Other studies have also attempted to look for a relationship between sagittal balance and pain [11-14]. One of these studies assessed the radiographic sagittal balance and found that good sagittal balance on imaging was inconsistently associated with the quality of life [11]. Therefore, it is unlikely that one variable in a multifactorial problem can explain low back pain. Therefore, despite a weak relationship, the positive correlation of muscular imbalance and low back pain may serve as an important consideration in further research. Further investigation in assessing a causative relationship may provide targeted therapy to prevent the worsening of pain. Further studies can explore if there is a relation between functional asymmetry and progression or improvement of radiographic sagittal balance.

The odds ratio for total lateral load tolerance greater than 50% of ideal body weight in relation to pain is 0.44 with 95% confidence intervals that include the null value and therefore cannot reject the null hypothesis as shown in Table 2. The linear regression coefficient of -5.81 suggests that there is a correlation where those with low back pain are less likely to have an increased lateral load tolerance; however, the R^2^ value of 8% indicates a weak relationship. Therefore, there is unlikely an association between lateral load tolerance and back pain. This finding coincides with prior studies that suggest postural control is not associated with back pain [6-9]. 

The odds ratio for total anterior-posterior tolerance in relation to low back pain was unable to be assessed because there were no participants who had a total anterior-posterior load tolerance <50% of ideal body weight with pain. The linear regression model coefficient is -0.11 suggesting a correlation between increased low back pain with decreased total anterior-posterior load tolerance. However, the R^2^ value <1% indicates a weak relationship between the variables. This finding coincides with prior studies that suggest postural control is not associated with back pain [6-9].

This study was limited to assess for correlation and not association therefore back pain could be independent of muscular symmetry. The study is a qualitative investigation to determine if a sufficient correlation exists between variables to merit future studies to test for association between low back pain and muscular asymmetry. This study was confined to participants located at a medical school which limits the generalizability of correlation.

## Conclusions

Increased muscular asymmetry in the sagittal plane and lateral plane showed a trend toward increased levels of low back pain, however, there is a weak correlation. This is a correlation and not an association. Future studies to assess the relationship between muscular balance and low back pain are needed to determine if therapy can be targeted to improve muscular sagittal balance, which can improve symmetry and back pain.

## Figures and Tables

**Figure 5:**
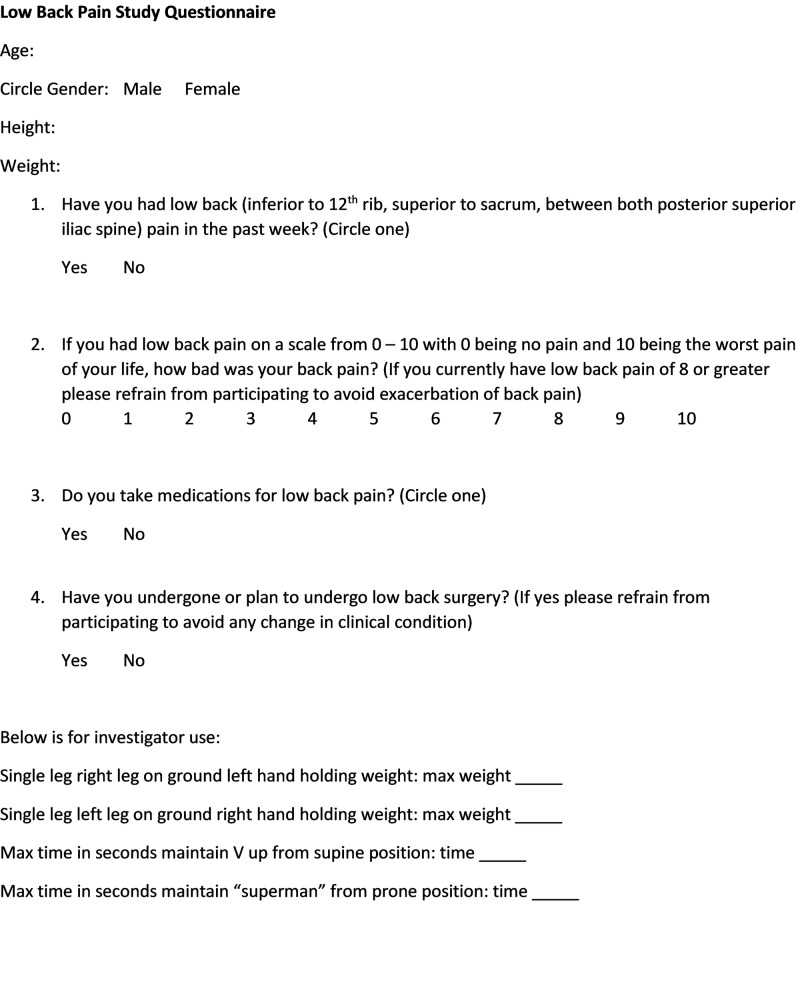
Participant questionnaire
